# Correlative Raman–Electron–Light (CREL) Microscopy Analysis of Lipid Droplets in Melanoma Cancer Stem Cells

**DOI:** 10.3390/bios12121102

**Published:** 2022-12-01

**Authors:** Francesca Pagliari, Elisa Sogne, Davide Panella, Gerardo Perozziello, Carlo Liberale, Gobind Das, Alice Turdo, Simone Di Franco, Joao Seco, Andrea Falqui, Santo Gratteri, Arturo Pujia, Enzo Di Fabrizio, Patrizio Candeloro, Luca Tirinato

**Affiliations:** 1 Division of Biomedical Physics in Radiation Oncology, German Cancer Research Center (DKFZ), 69120 Heidelberg, Germany; 2 Biological and Environmental Science and Engineering, King Abdullah University of Science and Technology (KAUST), Thuwal 23955-6900, Saudi Arabia; 3 PoliFAB, Polytechnic of Milan, Via Giuseppe Colombo, 81, 20133 Milan, Italy; 4 Nanotechnology Research Center, Department of Experimental and Clinical Medicine, University of Magna Graecia, 88100 Catanzaro, Italy; 5 Department of Physics, Khalifa University, Abu Dhabi P.O. Box 127788, United Arab Emirates; 6 Department of Health Promotion, Mother and Child Care, Internal Medicine and Medical Specialties (PROMISE), University of Palermo, 90127 Palermo, Italy; 7 Department of Surgical, Oncological and Stomatological Sciences (DICHIRONS), University of Palermo, 90127 Palermo, Italy; 8 Department of Physics and Astronomy, Heidelberg University, 69120 Heidelberg, Germany; 9 Department of Physics “Aldo Pontremoli”, University of Milan, Via Celoria 16, 20133 Milan, Italy; 10 Institute of Research for Food Safety & Health IRC-FSH, University Magna Graecia, 88100 Catanzaro, Italy; 11 Department of Medical and Surgical Science, University Magna Graecia, 88100 Catanzaro, Italy; 12 Department of Applied Science and Technology, Polytechnic of Turin, Corso Duca degli Abruzzi 24, 10129 Turin, Italy

**Keywords:** correlative microscopy, lipid droplets, melanoma cancer stem cells, Raman micro-imaging, electron microscopy

## Abstract

Among all neoplasms, melanoma is characterized by a very high percentage of cancer stem cells (CSCs). Several markers have been proposed for their identification, and lipid droplets (LDs) are among them. Different techniques are used for their characterization such as mass spectrometry, imaging techniques, and vibrational spectroscopies. Some emerging experimental approaches for the study of LDs are represented by correlative light–electron microscopy and by correlative Raman imaging–scanning electron microscopy (SEM). Based on these scientific approaches, we developed a novel methodology (CREL) by combining Raman micro-spectroscopy, confocal fluorescence microscopy, and SEM coupled with an energy-dispersive X-ray spectroscopy module. This procedure correlated cellular morphology, chemical properties, and spatial distribution from the same region of interest, and in this work, we presented the application of CREL for the analysis of LDs within patient-derived melanoma CSCs (MCSCs).

## 1. Introduction

Due to the high number of genetic aberrations that characterize its pathogenesis, with the activation of oncogenes or the inactivation of suppressor genes, melanoma is considered a highly heterogeneous disease. Despite the high degree of molecular heterogeneity and plasticity associated with melanoma cell mutations [1], this cancer type is characterized by the presence of a particularly high percentage of cancer stem cells (CSCs) [2,3]. This cell subset is believed to be responsible for tumor initiation, progression, and treatment failure [4]. Interestingly, melanoma CSCs are characterized by a larger lipid droplet (LD) content compared to their non-CSC counterparts [5]. In recent years, LDs have emerged as subcellular organelles involved in many physiological and pathophysiological functions, thus gaining significant attention from the research community [6,7,8,9,10]. 

Amid all parameters, the formation, amount, size, biochemical composition (both in lipids and in proteins), and distribution of LDs are the main aspects investigated by using different techniques such as fluorescence and electron microscopies, mass spectrometry, and Raman micro-spectroscopy. Despite the information derived by using one technique at a time, the ability to combine multiple assays would be highly desirable due to the amount of information that could be obtained and integrated, starting with a single specimen. The combination of light and electron microscopy allows for the study of specimens in a wide range of resolutions. Light microscopy can take images from broad areas at a lower resolution, and then electron microscopy can make images from specific regions at a much higher resolution. Light microscopy includes spectroscopic imaging techniques such as Raman micro-spectroscopy and fluorescence imaging. These methods enable the collecting of chemical information or the precise localization of areas with the high contrast offered by labeling, as is typical in fluorescence microscopy (FM) light microscopy. A very interesting approach that can serve this scope [11] is represented by correlative light and electron microscopy (CLEM). It combines FM and high-resolution electron microscopy (EM), collecting both functional information from the FM image and structural information from the EM image. Furthermore, the region of interest (ROI) can be selected using FM and then imaged at high resolution using EM. CLEM is indeed an ideal tool for investigating the complex relationship between form and function in biology [12,13].

A different combination of two techniques with the same purpose is the combination of correlative Raman and scanning electron microscopy (SEM) imaging [14,15,16,17]. In this setting, this approach takes advantage of the capabilities of Raman micro-spectroscopy to perform a molecular compositional analysis of a sample combined with the imaging of structural surface features at a high-resolution provided by SEM. 

Based on these scientific approaches, we developed a novel methodology by combining Raman micro-spectroscopy, confocal microscopy, and SEM coupled with an energy-dispersive X-ray spectroscopy (EDS) module, which we termed correlative Raman–electron–light (CREL) microscopy. The advantage of this further integration was that Raman micro-spectroscopy constitutes a label-free technique with a wide biochemical output, since it provides information all at once on several different classes of biomolecules, such as lipids, proteins, nucleotides, and metabolites. Conversely, fluorescence microscopy has a very limited biochemical output since it targets fewer biomolecules (according to the number of dyes used), but it provides more specific and quantitative information. Finally, electron microscopy provides structural information with the highest spatial resolution compared to Raman and fluorescence analyses, which are both limited optical techniques. As an example, within this framework, a cellomic experiment could be designed with fluorescent dyes being used to investigate specific biological pathways or the expression of specific biomolecules, coupled with Raman micro-imaging for assessing the overall biochemical behavior of the cells and with electron microscopy for obtaining a highly resolved morphology of the cells themselves. In the present work, we applied this technique to characterize cellular LDs within patient-derived melanoma CSCs (MCSCs) in a proof-of-principle experiment, correlating the cellular morphology, chemical properties, and spatial distribution from the same region of interest (ROI). In this case, fluorescence microscopy allowed for a unique and unambiguous identification of LDs, and their composition was further detailed by Raman micro-spectroscopy, while a highly resolved morphology was achieved using electron microscopy. 

## 2. Materials and Methods

### 2.1. Patterned Coverslips Preparation

Gold-patterned substrates, suitable for correlative light (Raman confocal) and SEM microscopy, were realized as described elsewhere [18]. Briefly, CaF_2_ microscope slides with diameters of 25 mm (Crystran Ltd., Poole, UK) were cleaned first in ethanol (Sigma-Aldrich Co., St. Louis, MI, USA) and then in 2-propanol (Sigma-Aldrich Co.) and finally dried with nitrogen (N_2_). After being put into a slide holder with a laser-cut stencil mask reproducing the desired pattern, they were loaded into a Cressington 208-HR (Cressington Scientific Instruments, Watford, UK) sputter coater under vacuum conditions equipped with a pure gold target (TedPella, Redding, CA, USA), and 70 nm of Au was deposited on the substrates.

### 2.2. Patient-Derived Melanoma Cancer Stem Cell Isolation and Characterization

MCSCs were isolated and cultured as previously described [19] following the ethical standards on human experimentation. Melanoma stem cells were propagated in ultra-low-attachment flasks (Corning Incorporated, Corning, New York, NY, USA) in a serum-free medium containing epidermal growth factor (EGF 20 ng/mL, PeproTech, London, UK) and basic fibroblast growth factor (βFGF 10 ng/mL, PeproTech) allowing the growth of melanoma stem cells as spheres.

MCSCs were routinely authenticated using the short tandem repeat system (GlobalFilter STR Kit, Applied Biosystems, Waltham, MA, USA) and subsequent DNA sequencing (ABIPRISM 3130 genetic analyzer, Applied Biosystems). Cells were constantly analyzed by a MycoAlert PLUS Mycoplasma Detection Kit (Lonza, Basel, Switzerland) to check for mycoplasma infection. 

### 2.3. Flow Cytometry

MCSCs were stained for ABCB5 (DyLight 488, NBP1-77689G, Novus Biologicals, Toronto, ON, USA). An isotype-matched control (IMC) was used as a negative control. Cells were incubated on ice with fluorescent-labeled primary antibody for 45 min on ice and subsequently washed with PBS and resuspended in FACS Flow. Data were acquired using a FACS ARIA I flow cytometer (BD Biosciences, Franklin Lakes, NJ, USA) and analyzed using FlowJo software analysis.

### 2.4. Raman Measurements

MCSCs were seeded on a patterned and sterilized CaF_2_ substrate in a CSC medium. After 24 h, the medium was replaced with pre-warmed HBSS (Thermo Fisher Scientific, Waltham, MA, USA) to avoid cell detachment from the substrate. Raman micro-spectroscopy was carried out using a Witec alpha300-RA. The excitation wavelength was 532 nm, and the incident light was focused on the sample through an Olympus 60×/1.0NA water immersion objective. The laser power at the sample level was about 2.0 mW. For the imaging experiments, cells were scanned through a laser focus in a raster pattern with a typical step size of 350 nm. Raman spectra were recorded in the 400–3.400 cm^−1^ spectral range, and the integration time was 0.2 s per pixel. With these parameters, about 1 h was required for scanning an area of 47 × 47 µm^2^, while the Raman mapping presented in this work took roughly 80 min. 

### 2.5. Raman Spectra Processing

The collected Raman spectra were pre-processed before multivariate analysis. First, the background spectrum of the HBSS medium was calculated as the average spectrum of all the regions where cells were not present on the map. Subsequently, this background spectrum was subtracted from all the Raman spectra, and further fluorescent effects were removed by the subtraction of a polynomial curve. Finally, all spectra in the dataset were normalized to the maximum spectral area recorded over the map. We did not normalize each single spectrum to its own area (which is a widely used normalization step) due to the small Raman signal recorded on many of the pixels located in the peripheral areas of the cells. Indeed, for all these pixels, which were affected by a small signal-to-noise ratio in the fast-mapping of cells, normalization to the small area under the spectrum curve would have resulted in an undesirable increase in noise. The Raman spectra resulting from these pre-processing steps were as close as possible to pure Raman scattering from the biochemical species within the cells without interferences from medium and cell autofluorescence. The whole Raman dataset was treated with multivariate tools, such as principal component analysis (PCA) and K-means clustering analysis (KCA), to extract biochemical features with particular attention paid to lipidomic issues. All Raman data analyses, both pre-processing and multivariate, were performed by using the free software package Raman Tool Set, freely available at http://ramantoolset.sourceforge.net/ (accessed on 17 January 2022) [20].

### 2.6. Confocal Sample Preparation and Imaging

Soon after the Raman measurements, MCSCs were fixed in 3% PFA and stained for nuclei and LDs with 1 μg/mL Hoechst 33342 (Thermo Fisher Scientific) and 0.1 μg/mL LD540 (Enamine, Kyiv, Ukraine) [21] dyes, respectively. Stained cells were washed three times with HBSS and resuspended in the same buffer. MCSCs were imaged using an upright Zeiss LSM 710 confocal microscope system equipped with a water immersion objective, C-apochromatic 40×/1.2 (NA 1.2). 

### 2.7. SEM Sample Preparation and Imaging

MCSCs were processed for SEM imaging immediately after the acquisition of the confocal images. They were fixed with glutaraldehyde 1% in sodium cacodylate 0.1 M for 20 min, washed three times with sodium cacodylate 0.1 M for 10 min, and post-fixed with 1% osmium tetroxide (OsO_4_) in sodium cacodylate 0.1 M for 20 min. After removing the OsO_4_ solution and rinsing twice with bi-distilled water, the sample was gradually dehydrated at room temperature by employing an ethanol series and then dried using hexamethyldisilane (HMDS). All the reagents were purchased from electron microscopy Sciences (EMS, Hatfield, PA, USA). Once dried, MCSCs were sputtered with a 15 nm gold layer (Cressington 208HR), and the images were acquired using a Zeiss Merlin scanning electron microscope, equipped with a Schottky field emission gun. The SEM images of the samples were acquired by collecting both the secondary electron (SE) and backscattered electron (BSE) signals, with the microscope working at an acceleration voltage of 20 kV, a beam current of 300 pA, and with an Everhart–Thornley detector, i.e., an in-chamber BSE and in-lens SE detector, respectively.

## 3. Results and Discussion

### 3.1. CREL Workflow

The ability to integrate several imaging techniques to investigate biological samples has allowed researchers to gain an increasing amount of information at one time. Depending on the nature of each technique, it may unveil morphological, structural, chemical, and many other crucial characteristics. In the present work, we combined three different techniques (Raman micro-spectroscopy, confocal, and SEM microscopies) to gain as much information as possible about the cellular LD content, their spatial distribution, and their composition inside MCSCs. Nowadays, determining the biochemical composition, amount, localization, and size of LDs is considered a pivotal piece of information in CSC biology. 

**Figure 1:**
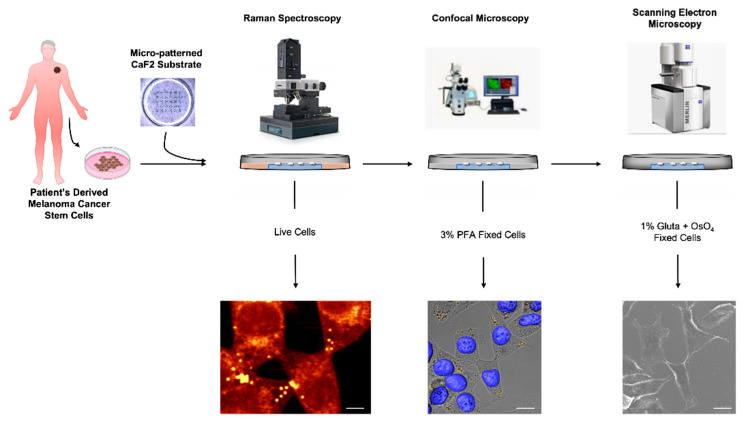
Representative scheme of the experimental design. Patient-derived MCSCs seeded on micro-patterned CaF_2_ substrates and analyzed by Raman spectroscopy in live conditions. After Raman measurements, MCSCs were fixed in 3% PFA and stained with LD540 and Hoechst33342 for LDs and nuclei, respectively. Lastly, PFA-fixed MCSCs were fixed again in 1% glutaraldehyde and OsO_4_ for SEM analysis.

We set up a simple sample preparation protocol, which was ideal for the different imaging analyses. The whole experimental workflow is reported in Figure 1.

The cells were kept alive for carrying out the Raman measurements and then fixed (with PFA and soon after with glutaraldehyde) for the confocal and electron microscopy analyses. 

First, the choice of an appropriate substrate was of primary importance in order to avoid interference with the results. The ideal substrate for Raman spectroscopic analysis is calcium fluoride (CaF_2_). In order to speed up the mapping of several ROIs and analyze the same cells in that region through the different imaging techniques, a reference micro-pattern was realized on CaF_2_ substrates. An array of 150 × 150 µm squares with the same size of field of view as the 20× objective of the optical microscope was fabricated in the center of the CaF_2_ substrates. 

### 3.2. Lipid Droplet Analysis by Raman Spectroscopy

Live MCSCs (Supporting Figure S1) seeded on CaF_2_ substrates were analyzed by Raman spectroscopy soon after removing them from the incubator. The cell medium was replaced with pre-warmed (37 °C) HBSS. 

**Figure 2:**
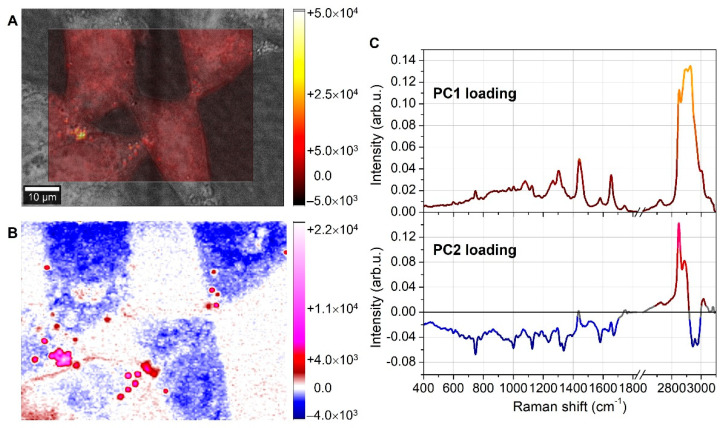
PC1 and PC2 score maps. Principal components PC1 and PC2 score maps, respectively, as shown in (**A**,**B**). Image (**C**) shows the PC1 and PC2 loading curves. PC1 loading curve (top of (**C**)) resembles the average spectrum recorded on the cells, and for this reason, PC1 score map (**A**) mainly accounted for topographical information. Instead, the PC2 loading curve (bottom (**C**)) exhibited strong positive lipid features at 2850 and 2885 cm^−1^, thus marking the lipid content. The corresponding PC2 score map (**B**) clearly showed the lipid droplet locations as bright-red-to-magenta regions. The colors of the loading curves in (**C**) reflected the color bars of the corresponding score maps in (**A**,**B**).

The PCA analysis was carried out on the Raman spectra, and the principal components (PCs) score maps along with the loading curves are reported in Figure 2 for the first two PCs, namely PC1 and PC2.

The loading curve of the first principal component (PC1) strongly resembled the average spectrum of the whole Raman dataset. This meant that PC1 mainly took into account pixel-to-pixel differences due to variations in the overall signal intensity. Consequently, the PC1 score map reflected the topographical information of the cells, where the highest signals were typically recorded where the cell thickness was larger. In addition, higher signals occurred where the biochemical species inside the cells had larger Raman scattering cross-sections. Indeed, the brightest regions of the PC1 score map were not located in the middle of the cell (nuclei), where the thickness was expected to be larger, but in some narrow spots of the external membrane. The PC2 loading curve and score map helped to identify the biochemical content of these narrow spots. As shown by the bottom curve of Figure 2C, the PC2 loading exhibited two strong positive peaks at 2850 and 2885 cm^−1^, which were well-known lipid Raman markers arising from CH_2_ stretching vibrations [22]. So, the bright red and magenta spots of the PC2 score map, corresponding to the brightest spots of the PC1 map, were narrow regions very rich in lipids, which were identified as LDs.

**Figure 3:**
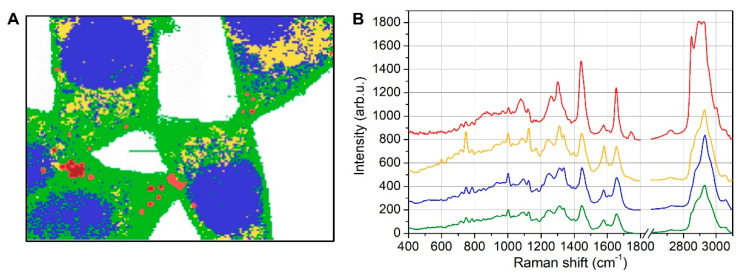
KCA map and average spectra. Resulting clusters obtained by KCA (**A**) and corresponding average spectra (**B**) (for clarity’s sake, the curves were shifted along the vertical axis). In (**A**), the blue areas were characterized by nucleotide features in the average spectrum, thus marking the nuclear regions along with ribosome spots in the cytoplasm; the yellow curve exhibits spectral features strongly related to Cyt C, and the corresponding regions in the map were classified as mitochondria areas; the red localized spots show strong Raman markers for lipids and were assigned to lipid droplets because of their spatial confinement.

Further investigations were performed by K-means clustering analysis, whose results are shown in Figure 3. 

Excluding the medium surrounding the cells (white areas in Figure 3A), five classes were used for the KCA analysis. Increasing the number of classes to six (and seven) produced an irregular fragmentation of blue (and green) areas. This further division obtained with six (and seven) classes was useless from a spectral point of view because they were characterized by average spectra that was fully similar to those obtained with five classes. In Figure 3A, the blue regions clearly identified nuclei, as evidenced by the corresponding average spectrum (blue curve in Figure 3B), which exhibited nucleotides and DNA-related peaks at 782, 1093, 1338, and 1578 cm^−1^ that were not observable in the other curves. The red and dark-red narrow spots in the clustering map were characterized by an average spectrum (red curve) dominated by lipid Raman features at 1076, 1262, 1301, 1440, 1654, 1743, 2850, and 2885 cm^−1^. The yellow regions showed distinctive spectral marks at 750, 1128, 1312, and 1582 cm^−1^ that were typical of cytochrome-C (CytC) [23,24], and thus were imputable to mitochondria. However, it is worth noticing that CytC undergoes a strong resonant Raman scattering when irradiated with a green laser (532 nm in the present case) due to the broad absorption band of CytC at 520 nm [25]. For this reason, the Raman signal from mitochondria was overwhelming the signal from other cellular organelles and subunits outside the nucleus. Some diffuse blue spots were also observed in the cytoplasmatic region and were ascribed to RNA nucleotides within ribosomes.

In the following section, to deepen the analysis of the LDs, we focused our attention on lipid signals, while the other peak assignments are briefly summarized in Supporting Table S1. The Raman peaks at both 1262 cm^−1^, which was due to =C–H cis stretching, and 1654 cm^−1^, which was due to C=C stretching, were undoubtedly indicators of unsaturated lipid chains [26]. It is also reasonable to hypothesize that the fatty acids were mostly mono-unsaturated due to the presence of the peak at 1301 cm^−1^ (CH_2_ twist) with an intensity higher than the 1262 cm^−1^ peak [27]. The broad band observable at 1076 cm^−1^ was due to C–C gauche stretching, and gauche conformations mainly occur in the more disordered fluid phase of lipids [27,28]. All the above considerations concerned the average spectrum recorded for the LDs (red curve of Figure 3B).

**Figure 4:**
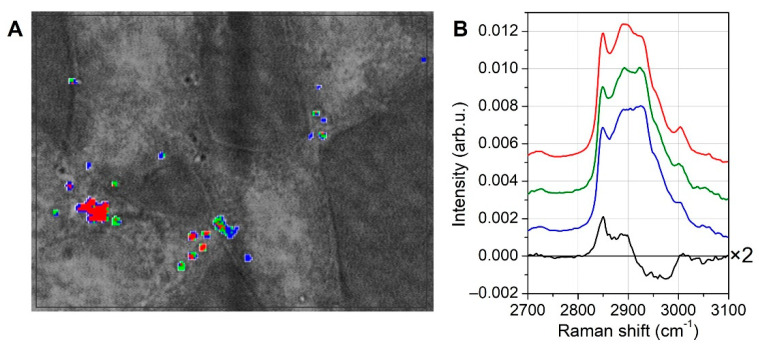
Analysis of lipid droplets. Only the spectra corresponding to lipid droplets were extracted from the full map dataset, and KCA was performed on this smaller lipid dataset. KCA clusters are shown in (**A**) and superimposed to the optical picture of the cells, while the corresponding average curves are shown in (**B**) (for clarity’s sake, the curves were shifted along the vertical axis). Since the fingerprint region was very similar for all the curves, in (**B**) we only showed the high-frequency region, where some small differences are observable. The black curve is the difference spectrum of red minus blue curve, indicating that lipid markers at 2850 and 2885 cm^−1^ were more pronounced in red regions.

To highlight the potential differences between the different LDs, we extracted only the spectra corresponding to lipid pixels from the whole map dataset, named the lipid dataset in the following section. The lipid dataset underwent KCA analysis and, since the number of spectra was much lower than the full map, only three classes were used for the calculation. The results are shown in Figure 4, where the clustered lipid pixels are superimposed to the bright field image (in Figure 4A), and the corresponding average spectra are reported for the high-frequency spectral region (in Figure 4B).

Figure 4B only shows the high-frequency spectral range, because in the fingerprint interval (400–1800 cm^−1^), the curves closely overlayed each other with no significant differences, and they resembled the average lipid spectrum recorded on the full map (red curve of Figure 3B). It is noteworthy that the colors used in this KCA analysis were not related to those formerly chosen for the full map analysis of Figure 3. In Figure 4A, it is possible to see that the major lipid spots were mainly dominated by red or blue color, and the corresponding average spectra are shown in Figure 4B. For highlighting the spectral differences between red and blue pixels, the difference curve of the red spectrum minus the blue spectrum is also shown in Figure 4B. Both peaks at 2850 and 2885 cm^−1^ were overexpressed in the red lipid spots compared to the blue ones, along with a more pronounced shoulder at 3005 cm^−1^. Since the curves in the fingerprint region were nearly the same, it was hard to decipher these differences in the high-frequency region. Even if red and blue lipid spots were again mostly mono-unsaturated fatty acids, because of the Raman peaks and relative intensities observed in the fingerprint region, two possible explanations for the small differences observed at high frequencies were proposed. First, these differences could be representative of the slightly different melting temperatures of the constituent fatty acids (which were, however, below room temperature in both the red and blue classes), and in turn this caused tiny differences in the conformation state of the fatty acids, where the lower the melting temperature, the higher the disorder. Alternatively, it was hypothesized that the Raman signal for the smallest droplets (which were blue) were partly affected by the neighboring pixels, and that their differences from the red ones should be ascribed to their surroundings. In addition, Figure 4A, along with the smallest blue LDs, also shows several blue and red droplets of comparable sizes, thus supporting the idea that the spectral differences were not simply size-dependent. In addition, it is worth remarking again that the low-frequency region did not exhibit any significant difference, and that an influence of neighboring pixels on the droplet signal would have also affected the low-frequency region. For these reasons, we believe that the first explanation was the most reliable, and that the observed slight differences could be ascribed to tiny differences in the composition of the LDs.

Altogether, these observations illustrated the feasibility of using Raman spectroscopy for analyzing general lipid content and, more in detail, the lipid composition of every single LD with a high spatial sensitivity. 

### 3.3. Lipid Droplet Analysis by Confocal Microscopy

**Figure 5:**
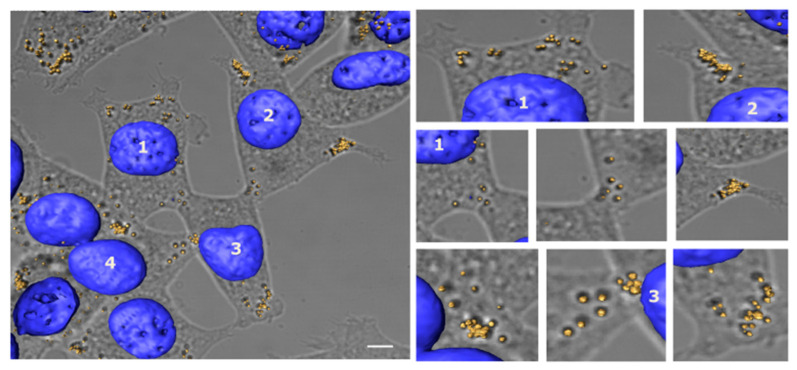
Confocal image of MCSCs stained for LDs and nuclei. Three-dimensional reconstruction of LDs (yellow) stained with LD540 and nuclei (blue) stained with Hoechst33342 overlapped with bright field max intensity projection of the relative z-stack images used for the reconstruction (scale bar, 10 μm). In the right panel, LD details are reported.

Soon after the Raman measurements were conducted, the live MCSCs were fixed in 3% PFA and stained for LDs and nuclei using LD540 and Hoeshct33342 dyes, respectively. The image reconstruction software Avizo 9.3 was used to process confocal image z-stacks with a step size of 0.67 µm. Background noise was reduced using Avizo’s Noise Reduction Median algorithms. Semi-automated image segmentation was used to extract and reconstruct the nuclei and LDs. By following the micro-pattern on the substrates, the ROI was quickly and easily identified (Figure 5). 

The images obtained clearly demonstrated that the fluorescently labeled LDs correlated with the corresponding Raman spots, thus confirming that the Raman signals were bona fide LDs. Moreover, since LDs are highly dynamic organelles whose positioning responds to cellular needs and stresses at the time of their acquisition [29,30], the correlation between the fixed confocal and live Raman images demonstrated that the methodology used here was accurate and fast enough to not induce subcellular LD redistribution. It is interesting to note that most of the LDs were mainly localized peripherally, with LD accumulation occurring in the proximity of cellular filopodia and cell–cell connections.

### 3.4. Lipid Droplet Analysis by Scanning Electron Microscopy

**Figure 6:**
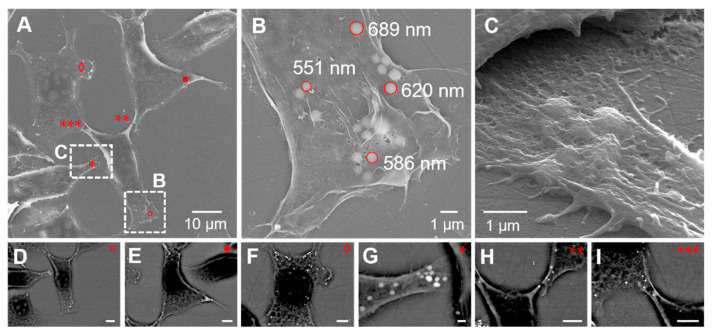
Scanning electron microscopy images of MCSCs. (**A**) SE image of the same ROI investigated by Raman and confocal microscopy; (**B**) detail of an MCSC area in which some LDs are identified and measured; (**C**) tilted image of part of MCSC with LDs; (**D**–**I**) BSE images of cells rich in LDs in the ROI. The areas of panels B and C are indicated in panel A, and the positions of panels (**D**–**I**) are also indicated in panel A by the corresponding symbols. Scale bars for panel (**D**–**I**) are all 5.0 um.

Once the confocal images were acquired, the MCSCs were prepared for SEM analysis as detailed in the “Materials and Methods” section. SEM with SE emissions is useful for studying the architecture of MCSCs, but the information obtained is limited to the cell surface. On the other hand, backscattered electron (BSE) emission can provide a high-resolution image of the specimen’s intracellular structure after heavy metal staining. In this study, we used BSE imaging analysis to investigate the arrangement of LDs in the MCSCs. The high-resolution SE and BSE–SEM images obtained are reported in Figure 6.

It is well known that possible shrinkage is usually generated during EM sample preparation. In particular, the chemical drying step using HMDS is the most critical one, resulting in morphological changes and possible different localizations of the targeted structures and, as a consequence, resulting in image misalignments in correlative imaging procedures [31,32]. Thus, although a slight decrease in cell volume was observed in the SE–SEM imaging, the images overall clearly showed that the LDs were preserved both in terms of numbers and in their cellular position. Furthermore, the mean higher Z-contrast provided by the BSE–SEM signal and observed in the same locations imaged by Raman and confocal imaging was likely due to the increased amount of osmium captured by the lipids contained in the droplets, thus further corroborating the spatial arrangement of the LDs.

## 4. Conclusions

In the present study, we described a novel correlative workflow (CREL) that integrated chemical, morphological, and spatial data from MCSCs, enabling us to gain information on LDs at the single LD level. Raman spectroscopy allowed us to perform an analysis of non-fixed and unlabelled cells, while the confocal analysis gave us an overview of the localization and distribution of LDs within cells. This was further corroborated by SEM analysis, which enriched the amount of data and provided us with structural surface details at a very high resolution, LD sizes, and an osmium-related higher BSE–SEM contrast as an indirect further indication of the presence of LDs (Supporting Figure S2).

The CREL approach could be applied to many different cell types and may be translated into the study of more complex biological problems where inter- and intra-cellular lipid variability (at the single-cell level) play a crucial role. This approach might help in adding new pieces of information toward the understanding of LD biology and its involvement in disease initiation and maintenance.

The CREL methodology could potentially be expanded by integrating further techniques (AFM, STED, FIB–SEM, X-ray, etc.) and by developing a more sophisticated image-processing system, especially for Raman micro-spectroscopy. Thanks to the development of new Raman tags (Raman dots, isotope labelled molecules, etc.), CREL could be implemented to also obtain a spatial mapping of both metabolites as well as multiple cellular targets within single cells. We expect that the recently developed coherent Raman micro-spectroscopy techniques, characterized by their very high spectral acquisition speed, detection sensitivity, spatial resolution, and penetration depth [33], will further boost the potential of the here-proposed correlative approach. Indeed, the CREL approach could be a promising protocol to also perform lipidomics on other cellular organelles such as mitochondria, lysosomes, the endoplasmatic reticulum, and the Golgi apparatus in different fields such as cancer biology, metabolic diseases, heart science, and neuroscience [34].

## Data Availability

All data generated are included in this study and in its supplementary file.

## Supplementary Materials

The following supporting information can be downloaded at: https://www.mdpi.com/article/10.3390/bios12121102/s1, Figure S1: Patient-derived melanoma cancer stem cells; Figure S2: Osmium BSE–SEM detection within LDs; Table S1: Tentative assignment of main Raman peaks.
