# Supplementary material for: Correlative Raman–Electron–Light (CREL) Microscopy Analysis of Lipid Droplets in Melanoma Cancer Stem Cells
Source: Biosensors (Basel). 2022 Dec 1;12(12):1102. doi: 10.3390/bios12121102 (PMC9776032; doi:10.3390/bios12121102)
Supplement: Supplementary file 1 [file biosensors-12-01102-s001.zip › biosensors-2019390-supplementary.pdf]

Supporting Information

Correlative Raman-Electron-Light (CREL) Microscopy Analysis of Lipid Droplets in Melanoma Cancer Stem Cells

Francesca Pagliari[1,2#], Elisa Sogne[2,3#], Davide Panella[4], Gerardo Perozziello[4], Carlo Liberale[2], Gobind Das[2,5], Alice Turdo[6], Simone Di Franco[7], Joao Seco[1,8], Andrea Falqui[9], Santo Gratteri[10], Arturo Pujia[11], Enzo Di Fabrizio[12], Patrizio Candeloro[4*] and Luca Tirinato[1,2,11*].

[1] Division of Biomedical Physics in Radiation Oncology, German Cancer Research Center (DKFZ), 69120 Heidelberg, Germany
[2] Biological and Environmental Science and Engineering, King Abdullah University of Science and Technology (KAUST), Saudi Arabia
[3] PoliFAB, Polytechnic of Milan, Via Giuseppe Colombo, 81, 20133 Milan, Italy
[4] Nanotechnology Research Center, Department of Experimental and Clinical Medicine, University of Magna Graecia, 88100 Catanzaro, Italy
[5] Department of Physics, Khalifa University, Abu Dhabi, United Arab Emirates
[6] Department of Health Promotion, Mother and Child Care, Internal Medicine, and Medical Specialties (PROMISE), University of Palermo, Palermo, Italy
[7] Department of Surgical, Oncological and Stomatological Sciences (DICHIRONS), University of Palermo, Palermo, Italy
[8] Department of Physics and Astronomy, Heidelberg University, 69120 Heidelberg, Germany
[9] Department of Physics "Aldo Pontremoli", University of Milan, Via Celoria 16, 20133 Milan, Italy
[10] Institute of Research for Food Safety & Health IRC-FSH, University Magna Graecia, 88100 Catanzaro, Italy
[11] Department of Medical and Surgical Science, University Magna Graecia, 88100, Catanzaro, Italy
[12] Department of Applied Science and Technology, Polytechnic of Turin, Corso Duca degli Abruzzi 24, 10129 Turin, Italy
# These authors contributed equally
* Correspondence: tirinato@unicz.it; patrizio.candeloro@unicz.it

Supporting Figure S1

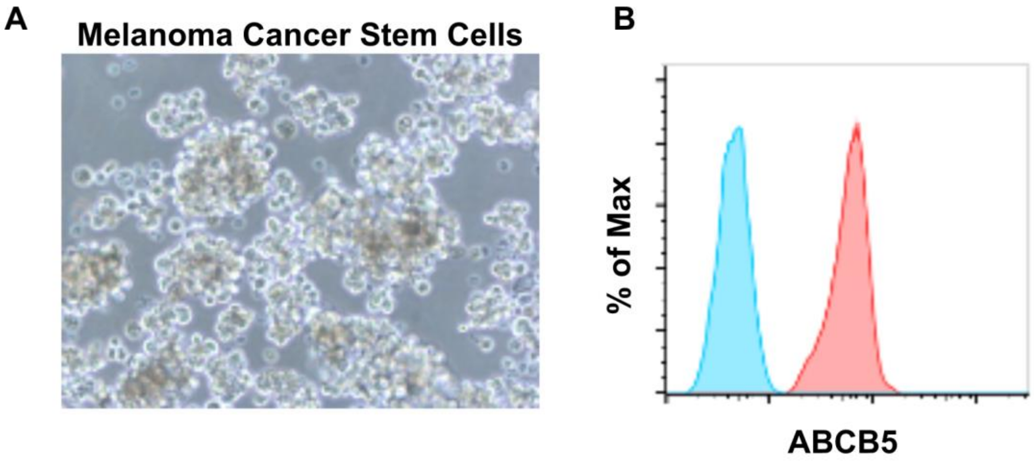

Figure S1. Patient-derived Melanoma Cancer Stem Cells. A) Representative phase contrast analysis of melanoma stem cells. **B)** Flow cytometry analysis of ABCB5 on melanoma stem cell. The isotype-matched control is shown in light blue.

Supporting Table S1

Raman Shift (cm-1)	Peak Assignment
750	Cytochrome C
782	**DNA/RNA** Nucleotides
1076	Stretching P-O-CH3
1093	DNA Backbone
1128	Cytochrome C
1262	C-H *cis* -stretch: **Unsaturated Lipids**
1301	CH2 Twist: **Saturated Lipids**
1312	CH3CH2 Twist: **Lipids**
1338	CH2/CH3 Wagging, Twisting &/or Bending mode: **Lipids**
1440	**Lipid/Protein** Marker
1578	G, A **DNA** Bases
1582	Cytochrome C
1654	Overlapping of Amide III with **Unsaturated Lipids**
1743	**Lipids**: Ester Band
2850	CH2 Symmetric Stretching: **Unsaturated Lipids**
2885	CH2 Asymmetric Stretching: **Saturated Lipids**

Table S1. Tentative assignment of main Raman peaks.

Supporting Figure S2

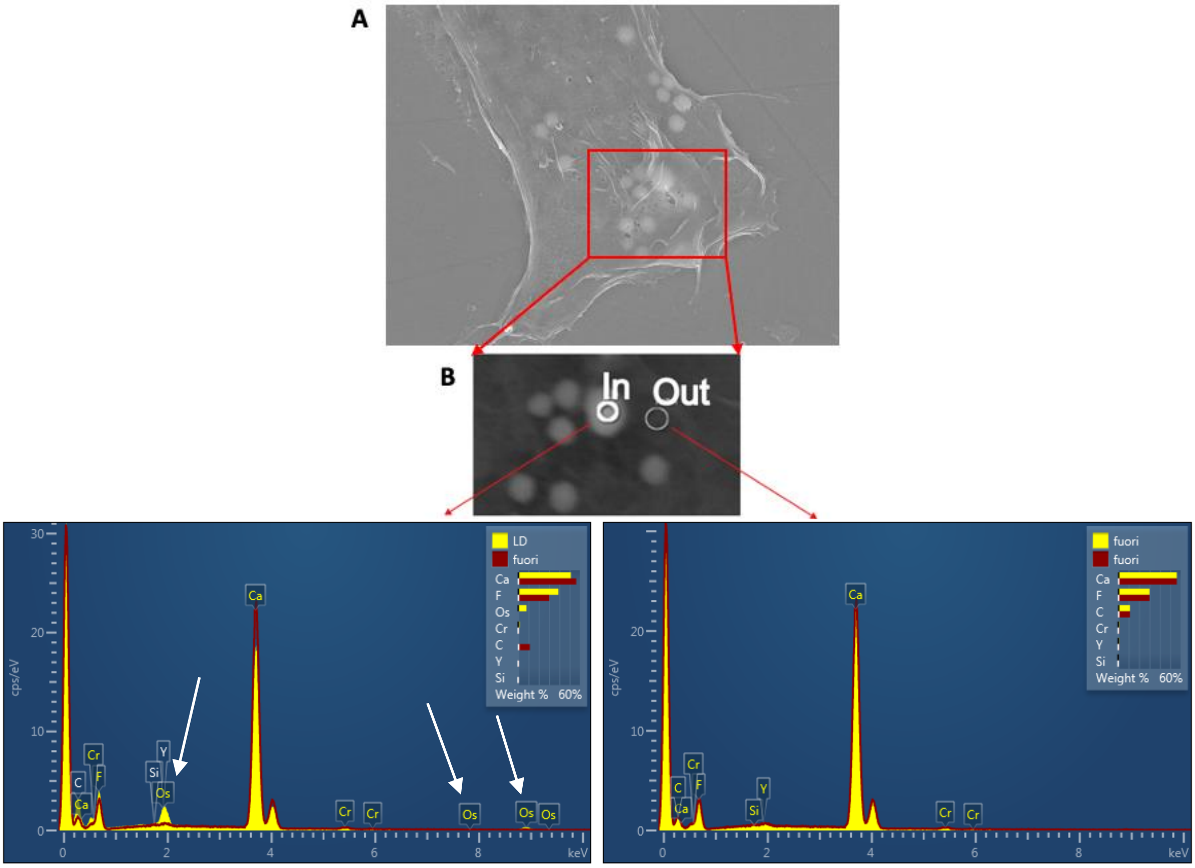

Figure S2. Osmium BSE-SEM detection within LDs. Osmium-related higher BSE-SEM contrast as an indirect further indication of LD presence
